# Inverse planned stereotactic intensity modulated radiotherapy (IMRT) in the treatment of incompletely and completely resected adenoid cystic carcinomas of the head and neck: initial clinical results and toxicity of treatment

**DOI:** 10.1186/1748-717X-1-17

**Published:** 2006-06-06

**Authors:** MW Münter, D Schulz-Ertner, H Hof, A Nikoghosyan, A Jensen, S Nill, P Huber, J Debus

**Affiliations:** 1 Department of Radiation Oncology, German Cancer Research Center (dkfz), Heidelberg, Germany; 2 Department of Medical Physics, German Cancer Research Center (dkfz), Heidelberg, Germany; 3 Department of Radiation Oncology, University of Heidelberg, Heidelberg, Germany

## Abstract

**Background:**

Presenting the initial clinical results in the treatment of complex shaped adenoid cystic carcinomas (ACC) of the head and neck region by inverse planned stereotactic IMRT.

**Materials:**

25 patients with huge ACC in different areas of the head and neck were treated. At the time of radiotherapy two patients already suffered from distant metastases. A complete resection of the tumor was possible in only 4 patients. The remaining patients were incompletely resected (R2: 20; R1: 1). 21 patients received an integrated boost IMRT (IBRT), which allow the use of different single doses for different target volumes in one fraction. All patients were treated after inverse treatment planning and stereotactic target point localization.

**Results:**

The mean folllow-up was 22.8 months (91 – 1490 days). According to Kaplan Meier the three year overall survival rate was 72%. 4 patients died caused by a systemic progression of the disease. The three-year recurrence free survival was according to Kaplan Meier in this group of patients 38%. 3 patients developed an in-field recurrence and 3 patient showed a metastasis in an adjacent lymph node of the head and neck region. One patient with an in-field recurrence and a patient with the lymph node recurrence could be re-treated by radiotherapy. Both patients are now controlled. Acute side effects >Grade II did only appear so far in a small number of patients.

**Conclusion:**

The inverse planned stereotactic IMRT is feasible in the treatment of ACC. By using IMRT, high control rates and low side effects could by achieved. Further evaluation concerning the long term follow-up is needed. Due to the technical advantage of IMRT this treatment modality should be used if a particle therapy is not available.

## Background

Adenoid cystic carcinomas of the head and neck are a unique kind of tumour deriving from cells of the major and minor salivary glands, they account for 25% of all malignant salivary gland tumours in different series. Although ACCs are the most common histological type of tumours in the minor salivary glands with a total of 55% [1] they account for only about 10–15% of all parotid gland malignancies. ACCs can be shown to be unique for various reasons: first of all, these tumours often show a protracted natural history. When compared to other malignancies, a prolonged survival of patients with ACCs is possible even in case of local recurrence or distant metastases. Second, ACCs characteristically exhibit a locally aggressive growth pattern with a unique tendency to nervous tissue invasion. They might also spread greater distances from the primary location by growing along the nerve sheaths. In some cases, skip involvement can be seen along the perineural space.

Hence, treatment of adenoid cystic carcinomas of the head and neck presents a serious therapeutic challenge in most cases. Surgical treatment is limited by the tumours' growth pattern mentioned above. Despite complete gross tumor resection due to paraneural spread in some cases surgery is not radical. However, surgery is still the preferred treatment of ACCs but complete resection might not always be possible or feasible if too many normal structures are injured.

So radiation therapy often is an important part in the treatment, either as postoperative therapy or as definite treatment if a complete resection of the tumour is not possible [2,3]. However, the radiotherapeutic approach is also similarly challenging due to numerous radiosensitive structures in the head and neck region as doses ranging between 60 and 70 Gy should be achieved in macroscopic tumours. At these total doses normal tissue reactions in curable disease is frequent and should be reduced by introducing new planning technologies. Recent advances in radiation oncology and treatment planning led to the implementation of inverse planned intensity modulated radiation therapy (IMRT). This treatment technique allows the optimisation of target volume coverage while allowing to reduce the dose to surrounding organs at risk. Therefore an increased total dose could be achieved with a better coverage of the target volume. Initial results using IMRT show an improvement of local tumour control rates and toxicity profiles in patients with tumours of the head and neck [4-6]. The aim of this publication is to present the inverse planned IMRT treatment technique, initial clinical results, and toxicities in patients with complex shaped ACCs of the head and neck.

## Methods

### Patients

25 patients with complex shaped ACCs were treated at our institution with inverse planned IMRT. Their age ranged between 30 and 80 years (median: 59 years) with 12 female and 13 male patients. The diagnosis of ACC was confirmed histologically for all patients. 18 patients had surgery aimed at total or subtotal resection of the tumour before radiation therapy, 7 patients only had a biopsy of the tumour for histological confirmation. According to pathology reports, tumours could be completely resected (R0) in 4 patients and with microscopic residuals (R1) in one patient. In the remaining patients, surgery could only achieve an R2 resection. At the time of radiation therapy, 2 patients already had lung metastases (M1), which showed no progression in CT for more than 3 months at the beginning of radiation therapy. Additionally, involvement of cervical lymph nodes was histologically confirmed in 2 patients. Only one patient received chemotherapy before radiation treatment. The anatomical location of the ACCs treated is displayed in table 1.

**Table 1:** Localization of the tumor

**Tumor Localization**	**Number of patients**
Base of skull/Sinus maxillaris	17
Glandula lacrimalis	2
Tongue	2
Orbita	1
Trachea	1
Oropharynx	1
Acustic meatus	1

### Immobilisation

Adequate immobilisation is a crucial prerequisite for obtaining the high accuracy needed for stereotactically guided IMRT and therefore an important part of the definition of IMRT at our institution. Hence, a customised individual immobilisation device depending on the tumour location was created for each individual patient. For treatment of lesions in the brain or base of skull the patient was positioned supine with an individually made mask which, at the German Cancer Research Centre (dkfz), consists of Scotch Cast material wrapped around the head of the patient. Serial 3 mm CT slices of the of the whole treatment area plus a superior margin of 5 cm and an adequate inferior margin to cover the neck lymph nodes level I-III were acquired using a stereotactic head frame. Contrast agent was administered according to our in-house protocol. Additionally, a contrast MRI scan was performed of the primary tumour area with the patient also positioned in the individual mask and using a stereotactic head frame.

### Target definition

Target volumes and organs at risk were defined slice by slice on the treatment planning CTs using the 3D-conformal radiotherapy planning system VIRTUOS which was developed at the dkfz. An image fusion of the treatment planning CT and the MRI scan was performed for a more accurate definition of the target volume and critical normal structures. Image registration was done stereotactically.

The CTV I was defined as the visible tumour volume in the imaging studies plus a margin of 2 mm. For patient treated after a complete resection the area of the tumours visualized by preoperative scans was defined as CTV I. CTV II was drawn around the CTV I with a generous margin depending on the anatomical relationship of adjacent structures and potential microscopic spread. Areas with potential subclinical disease, in particular the neurovascular sheaths in ACCs, were also included in the CTV II. The ipsilateral lymph node level direct adjacent to the primary tumor region was furthermore defined as CTV II in all cases. Additional lymph nodes or lymph node level highly suspicious for tumor in CT or MRI or after biopsy were defined as CTV II This was necessary for two patients. One of these two patients received treatment of bilateral lymph node levels II and III. Volume of CTV I ranged between 50 to 896 cm^3 ^(Mean: 257 cm^3^) and for CTV II between 233 and 950 cm^3 ^(Mean: 433 cm^3^). Surrounding organs at risk were also contoured slice by slice with special regard to the spinal cord, brainstem, optical nerve, chiasm, and the temporomandibular joint.

### Treatment planning

KonRad inverse planning system developed at the dkfz and now commercially available at Siemens Medical Solutions was used for inverse treatment planning and was linked to VIRTUOS for 3D dose calculation and visualisation. No additional margins were defined around the target volumes in the inverse treatment planning program.

In order to start the optimisation process in KonRad, the user needs to define maximum and minimum dose constraints on both the target volumes and the organs at risk. Moreover, penalties defining the relative importance of the individual constraints have to be specified. Based on these constraints and penalties, KonRad then uses an iterative optimisation algorithm (gradient technique) in order to improve the 3D dose distribution by minimising the objective function. In addition to the dose specifications, the user is also able to select the couch, gantry, and collimator angles as well as the number of intensity levels representing the complexity of beam modulation in the KonRad planning system. On optimisation, treatment plans using 5, 7, or 9 equidistant gantry angles, coplanar or non-coplanar couch position were evaluated in order to define the beam arrangement with the best target volume coverage and normal tissue sparing. 4 to 8 non-zero intensity levels were used to define the fluence map. An integrated boost radiotherapy (IBRT) was optionally used for patients with macroscopic tumors [16]. This IMRT technique allows the treatment of various target volumes simultaneously with various doses per fraction and in turn allows escalation of the individual dose per fraction to the CTV I.

The total doses for all target volumes were prescribed to the median of the target volumes with 50% of the individual target volume receiving 100% of the total dose. At our institution, the dose of all IMRT treatment plans is prescribed to the median.

The maximum dose to the cervical spinal cord was limited to 45 Gy while the maximum dose to the brainstem, the optic nerve and chiasm was limited to 54 Gy. For the remaining organs at risk the dose thresholds specified by [17] were accepted. The target point of the IMRT treatment was defined stereotactically and treatment was performed on a step-and-shoot approach using a linear accelerator with photon energies of 6 MV and 15 MV and integrated multi-leaf collimator (MLC) (Siemens Medical Solutions).

### Follow-Up

The patients were seen regularly for clinical examination during and after radiation therapy. Acute toxicity was specified according to the Common Toxicity Criteria (CTC) during treatment and 3 months afterwards. After 3 months, the RTOG/EORTC criteria for radiation late-effects were used for evaluation. The first MRI scan of the area treated was performed 6 weeks after radiation therapy was completed. For the first 2 years after radiation therapy, patients were followed up in intervals of 3 to 6 months including clinical examination and MRI scans. Depending on clinical and radiological findings the period between individual follow-ups was increased after the second year. The measurement of follow-up was initiated with the beginning of radiation therapy. The Kaplan-Meier method was applied to analysis of overall survival, disease-free survival, and control rate.

## Results

### Technical aspects

Integrated boost IMRT (IBRT) was performed in 21 patients. The median total dose delivered to the CTV I was 66 Gy (range: 58 – 70.4 Gy) and the median dose per fraction to the CTV I was 2 Gy (range 1.8 – 2.2 Gy). Only in one patient with an macroscopic ACC the dose of the CTV I was lower than 60 Gy due to a diffuse infiltrating tumor with a close relationship to the myelon and the brainstem. Using the same number of fractions, the median total dose to the CTV II was 60 Gy (range 56 – 64.2 Gy) and the median dose per fraction was 1.9 Gy (1.7 – 2.0 Gy). In only 2 patients a non-coplanar beam set-up was used. The median number of beams applied was 7 (range 5 – 9). The total number of sub-segments for the step-and-shoot IMRT approach ranged between 48 and 100 (median 75). Approximately 6 segments per minute could be applied by the system used. Therefore, the absolute treatment time ranged between 8 and 16.7 minutes (median: 12.5 minutes). For rotation of the gantry to the next treatment position approximately 8 sec. were needed. By two minutes increased the treatment time if a movement of the table for non-coplanar treatment was necessary. Additional 5 minutes were needed to position the patient in the mask and for target point localisation and adjustment.

### Outcome

**Figure 1:**
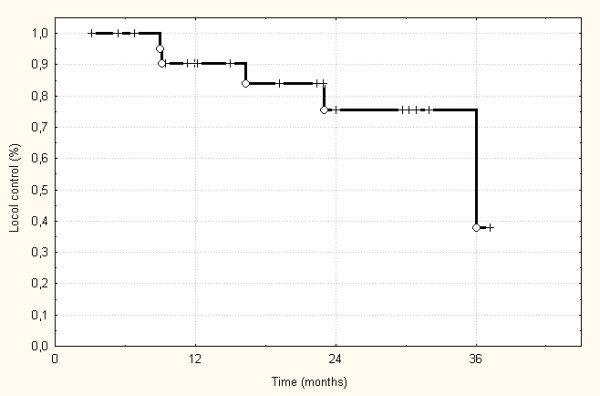
Loco-Regional control probability according to Kaplan-Meier.

**Figure 2:**
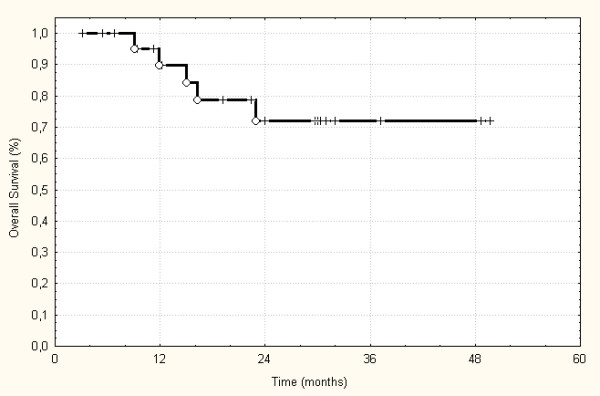
Actuarial overall survival of the treated 25 patients.

The treatment schedule could be finished for all patients as planned initially, and treatment interruptions were not necessary for any longer than 2 days. Patients were followed-up between 3 to 49.5 months (median 22.5 months). Radiological evaluation of the patients revealed complete remission in 2 patients (8%), partial remission in 6 patients (24%), and no change in the remaining patients according to the WHO definitions. None of the patients showed progression of disease under radiotherapy and on the first follow-up (after 6 weeks). 3 patients developed an in-field recurrence, and 3 patients had a progression of their disease in an adjacent lymph node. The actuarial local control rate was 38% after the three years (Figure 1). Additionally, 2 patients developed distant metastases while local control was achieved. According to Kaplan-Meier, the 3-year-progression-free-survival was 30%. One patient with in-field failure and one patient with neck node recurrence could be re-treated with IMRT. Both are controlled now. Including these 2 patients in the analysis of the 3-year-progression-free survival this is increased to 48% according to Kaplan-Meier. Unfortunately, 5 patients died of their disease caused by systemic progression in 2 patients and local failure in 3 patients. Therefore, the overall survival in this patient group was 72% after 3 years (Figure 2). Analyzing the 5 patients separately receiving treatment after R1 or R0 resection only one patients died caused by metastatic disease. All these patients were locally controlled until now.

### Treatment toxicity

Treatment had to be interrupted in only one patient for no longer than 2 days due to acute side-effects. Severe acute side-effects were mucositis grade III, seen in 5 patients (20%), and mucositis grade II, seen in 11 patients (44%). 1 patient (4%) showed a grade III skin reaction while 11 patients (44%) showed a grade II skin reaction. In addition, a slight hair loss, nausea, inner ear effusion, and xerostomia occurred during radiotherapy. Apart from the xerostomia, these symptoms had completely disappeared 3 months after completion of radiotherapy. Xerostomia grade II has so far been the only severe late-effect observed in 5 patients.

## Discussion

The data presented are able to support that IMRT is a safe and feasible modality in the treatment of complex-shaped ACCs in the head and neck. By using IMRT, high doses in the GTV and PTV can be achieved together with an excellent normal tissue sparing, especially bearing in mind the dose-response relationship for ACCs described by various authors. Garden et al. [7] could demonstrate significant differences with respect to local control rates between patients treated with doses of less than 56 Gy and patients treated with more than 56 Gy. Hence, these authors are recommending a dose of 60 Gy to the tumour bed and 66 Gy to the tumour bed in patients with positive margins. Harrison at al. [8] could show a comparable effect using doses of at least 57.5 Gy compared with lower doses for histologically different major salivary gland tumours. The authors found a reduced 10-year local control rate with 53% in patients treated with lower doses compared to 72% in patients treated with more than 57.5 Gy. Using high doses to the CTV I in this study, an in-field recurrence only occurred in 3 patients so far. Furthermore, IMRT offers the advantage of treating various target volumes with different doses per fraction. This results in a reduced overall treatment time and increased doses per fraction to areas with macroscopic disease. IBRT hence does also have advantages especially with respect to radiobiology. In this study, 21 out of the 25 patients were treated with IBRT showing no increase in side-effects when compared with the four patients treated with a conventional IMRT regimen. All in all, acute and late side-effects were negligible in this study so far. No grade IV toxicity was seen, and only 20% of the patients developed a grade III toxicity of the mucosa. These results are comparable to the results from a group of patients treated with photons in the study of Huber et al. [9]. This study demonstrated similar acute side-effects for patients treated with photons. Nevertheless, patients treated with neutrons showed increased late-effects when compared to patients receiving photon radiotherapy.

Indications have to be defined for IMRT in the treatment of ACCs when compared to radiotherapy regimen using high relative biological effectiveness (RBE) beams. With respect to high-RBE beams, the longest experience exists using neutrons. The use of neutrons compared to photons shows an advantage in most studies. The only prospective randomised trial comparing neutrons and photons [11] revealed an increased 2-year-survival rate with 62% in patients treated with neutrons compared to 25% patients treated with photons (p < 0.005). Additionally, the loco-regional control rates at two years were 67% for neutrons and 17% for photons (p = 0.10). The final report of this study with a follow-up of ten years and 25 patients supports the initially published results demonstrating significantly higher control rates in the treatment with neutrons without improvement of overall survival [13].

Douglas et al. [14] published a huge trial of 151 patients with non-resectable ACC treated with neutrons. The 5-year-actuarial local control rate was 57% and the actuarial overall survival was 72%. A retrospective trial by Huber et al. [9] compared the results of our institution in the treatment of ACCs to treatments with neutrons alone, with a combination of neutrons and photons, and photons alone. The local control rates were 75% for neutrons and 32% for the combined therapy as well as photons alone. No differences could be observed with respect to the overall survival in this study. Promising results in reduction of side effects and improvement of outcome were presented by our group when combining IMRT and carbon ion therapy as a boost treatment. In this prospective study published by Schulz-Ertner et al. [15] there was no higher late toxicity than CTC grade II so far. Additionally, the 3-year actuarial overall survival rate was 83.3%. Only 3 patients had a loco-regional recurrence at a median follow-up of 12 months.

## Conclusion

The data presented in our study shows comparable results to most studies using photon beams for the treatment of ACCs. IBRT and IMRT with the tested dose concept is feasible and results in a promising clinical outcome justifying further investigations. Furthermore describes the IBRT concept a promising approach to escalate the dose further while reducing the treatment time.

A prospective study investigating the possible advantages of IBRT plus carbon ion boost compared to IBRT alone needs to be performed. Nevertheless, long-term results in treatment of ACCs remain disappointing even applying highly sophisticated radio-oncological treatment techniques due to the high number of distant metastases. Hence, future therapy of ACCs should also strongly focus on improvement systemic treatment of this disease.

## Authors' contributions

MWM, DSE, HH, AN performed the patient study and treatment planning. Statistical analysis and patient follow-up was done by AJ, MWM, SN and PH. Manuscript was drafted by PH, MWM, JD. All authors read and approved final manuscript.
